# Use of IR Biotyper as a feasible methodology to type *Klebsiella pneumoniae*

**DOI:** 10.1128/spectrum.01146-25

**Published:** 2025-10-06

**Authors:** Patricia Orlandi Barth, Camila Mörschbächer Wilhelm, Dariane Castro Pereira, Laura Czekster Antochevis, Andreza Francisco Martins, Afonso Luís Barth

**Affiliations:** 1 Laboratório de Pesquisa em Resistência Bacteriana, Hospital de Clínicas de Porto Alegre, Porto Alegre, Rio Grande do Sul, Brazil; 2 Programa de Pós-Graduação em Ciências Médicas, Universidade Federal do Rio Grande do Sul, Porto Alegre, Rio Grande do Sul, Brazil; 3 Unidade de Microbiologia e Biologia Molecular, Hospital de Clínicas de Porto Alegre, Porto Alegre, Rio Grande do Sul, Brazil; 4 Programa de Pós-Graduação em Ciências Farmacêuticas, Universidade Federal do Rio Grande do Sul 28124, Porto Alegre, Rio Grande do Sul, Brazil; Ann & Robert H. Lurie Children's Hospital of Chicago, Chicago, Illinois, USA

**Keywords:** IR Biotyper, *Klebsiella pneumoniae*, KL

## Abstract

*Klebsiella pneumoniae* is one of the most frequently reported healthcare-associated pathogens. The current gold standard approach to perform the epidemiological typing of these bacteria is Whole Genome Sequencing (WGS), which is an expensive and challenging procedure. IR Biotyper (Bruker Daltonics, GmbH) is a new equipment based on Fourier transform infrared spectroscopy, which allows a rapid, low-cost, and user-friendly method to type bacterial isolates. However, there is a need for studies that evaluate the efficacy of the IR Biotyper. The aim of this study was to evaluate the capability of IR Biotyper to type *K. pneumoniae* according to sequence type (ST) and capsular type—using K locus (KL)—as well as to develop a classifier using machine learning. Seventy-three isolates of *K. pneumoniae* previously characterized by WGS were selected for IR Biotyper analysis using principal component analysis for dimensionality reduction, Euclidean, and unweighted pair group method with arithmetic mean (UPGMA) for clustering method, and spectra were analyzed in the 1,300–800 cm⁻¹ wavenumber range. Among these, 54 isolates were used to create a classifier, and 19 were used to validate the classifier. When considering the ST, ST307 was grouped in the same cluster as ST11. When KL was considered for the analysis, the clusters were 100% correctly grouped according to their KL type. Furthermore, the classifier developed was able to classify the isolates according to KL with a high concordance. This study showed that KL correlates well with KL for typing *K. pneumoniae* isolates using the IR Biotyper. Additionally, IR Biotyper demonstrated to be a cost-effective method and a promising tool to classify isolates within minutes.

**IMPORTANCE:**

*Klebsiella pneumoniae* is a major cause of severe hospital infections, and controlling its spread requires quick identification and comparison of bacterial strains. WGS is accurate but expensive, slow, and technically demanding. In this study, we evaluated the IR Biotyper, a device that uses infrared light to analyze bacteria and group them by capsule type—a key feature linked to their spread. The IR Biotyper matched WGS results with high accuracy, delivering results in minutes instead of days. This fast, affordable method can help hospitals detect outbreaks earlier and respond more effectively. Our findings suggest that the IR Biotyper is a valuable tool for routine use in microbiology laboratories, supporting epidemiological surveillance and outbreak control.

## INTRODUCTION

Carbapenem-resistant *Klebsiella pneumoniae* (CRKP) is one of the top priority pathogens of concern in public health (1). Therefore, it is important to develop methodologies to track in real time the spread in healthcare settings of the CRKP strains with high potential to cause outbreaks of infection due to increased antibiotic resistance and/or with virulence factors (2, 3).

Bacterial typing is commonly performed for monitoring and characterization of bacterial isolates by using genetic methods, which can determine capsular type according to the K locus (KL), strain type (ST), core genome multilocus sequence typing, and other features (4, 5). These methods, which demand sequencing of some genes or the whole genome, are expensive, laborious, and time-consuming. As an alternative, Fourier-transform infrared (FTIR) spectroscopy has emerged as a tool for typing (6, 7).

FTIR spectroscopy is a phenotypic method based on the differential vibration of distinct chemical bonds of the molecules present in the bacterial cell by exposure to an infrared beam. As distinct functional chemical groups absorb infrared radiation at different wavelengths, specific spectral peaks are generated, which are related to the absorbance to wavenumbers (8). This methodology has been applied to evaluate the relationship among isolates in an outbreak (9–11) and to classify bacteria according to a specific feature, such as the KL type (12, 13), which can be used for bacterial characterization and surveillance. The KL is a chromosomal region that encodes approximately 10–30 genes responsible for the capsule biosynthesis pathway. To date, there are a total of 147 loci defined, and the KL types were found to be very related to the serologically defined K type (capsule antigen) (14).

Although studies evaluating KL typing by FTIR have employed the mode of attenuated total reflectance (ATR) and presented promising results (12, 13), studies assessing the transmission mode are still lacking.

IR Biotyper is a platform developed by Bruker (Daltonik, GmbH), which uses FTIR in transmission mode and presents a very user-friendly software. It allows the users to evaluate the relatedness of bacterial isolates in only a few minutes and to classify them according to some features with the application of artificial intelligence to develop a classifier (7, 15). Therefore, the aim of this study was to develop a classifier and evaluate the capability of IR Biotyper in classifying bacterial isolates according to KL type. The study also evaluated the performance of IR Biotyper for typing *K. pneumoniae* according to the ST.

## MATERIALS AND METHODS

### Selection of isolates

A total of 73 *K*. *pneumoniae* previously characterized by Whole Genome Sequencing (WGS) (Illumina miSeq) as part of a routine epidemiological surveillance in our laboratory were selected for IR Biotyper analysis. The strains were collected between 2019 and 2023, representing a variety of clinically relevant STs and KLs frequently associated with healthcare-associated infections. The selection aimed to include genetic diversity (six STs and seven KLs) in order to build and validate a robust FT-IR classifier.

The isolates were randomly divided into a training set (*n* = 54) and a validation set (*n* = 19), containing the most prevalent STs in our country, while maintaining the proportional representation of different ST and KL types in both sets. The training set was used to build the KL-based classifier using the IR Biotyper software. The validation set, composed of isolates that were not included in the classifier training, was used to independently assess the classifier’s performance. This approach was used to avoid bias and to ensure that the validation set truly tested distinct isolates of the classifier.

Isolates that were phenotypically translucent on agar Müller-Hinton were excluded, as previous tests showed that the color of the colonies could affect the interpretation by IR Biotyper (Fig. S1).

### Whole genome sequencing

The isolates were sequenced by Illumina MiSeq (2 × 250 bp; average depth coverage ∼100×) using genomic DNA extracted by a Maxwell RSC Cultured Cells DNA Kit (Promega). The concentration was determined by Qubit dsDNA HS Assay Kit with a Qubit four fluorometer (Thermo Fisher Scientific) and fragment lengths assessed using the TapeStation 2200 (Agilent, UK). The quality of DNA was determined by NanoDrop, and the 260/280 ratio was considered. The paired-end library was constructed with Illumina DNA prep (Illumina) according to the manufacturer’s protocols. The raw short reads were quality trimmed (*Q* > 30) and assembled using CLC Genomic Workbench 23. Sequence types and KLocus were obtained from PathogenWatch, a bioinformatics tool used for WGS-based typing.

### IR Biotyper

The selected isolates were phenotypically characterized by FTIR spectroscopy methodology using the IR Biotyper (Bruker Daltonics) equipment. Bacterial isolates were subcultured onto MacConkey agar, and after growth, were subcultured onto Müeller-Hinton agar plates. A bacterial suspension for each isolate was prepared by resuspending a 1 µL loopful of bacterial colonies in 50 µL of 70% ethanol solution in an IR Biotyper suspension vial containing sterile metal rods. The suspensions were vigorously vortexed, and 50 µL of deionized water was added and vortexed again. Fifteen microliters of the bacterial suspension was spotted in five technical replicates onto the IR Biotyper target plate and left to dry in ambient air. Spectra were acquired using OPUS 7.5 software (Bruker Daltonics GmbH & Co. KG, Bremen, Germany). The spectrum range of 1,300–800 cm was automatically processed and normalized, and second derivatives were applied by the IR Biotyper software. The data were also analyzed with IR Biotyper software by initially verifying the distribution of the isolates with principal component analysis (PCA) for dimensionality reduction into 2D graphics and Euclidean and unweighted pair group method with arithmetic mean (UPGMA), a statistical method used by the software to calculate pairwise spectral distances and generate hierarchical clustering, for clustering method.

Then a classifier, a machine learning model included in the IR Biotyper software, was developed in order to classify the isolates according to the KL type, following the manufacturer’s instructions. Default splicing method and artificial neural network with previous PCA were applied with the following parameters: a cutoff value of 0.95 was applied to define spectral similarity between isolates, and the first 20 principal components (PCs) were included in the model. The similarity matrix was calculated based on the correlation of the PCA scores among the spectra, as per the manufacturer’s standard algorithm. The clustering was performed using the built-in hierarchical clustering algorithm provided by the IR Biotyper software, and no additional training cycles were required, as the classification is unsupervised and data driven. The outlier and inlier for result reliability thresholds were set at 4.99 and 4.98, respectively. The isolates selected for the training set are listed in Table S1. The remaining isolates used for the validation set are listed in Table S2.

## RESULTS

Among the 54 *K. pneumoniae* isolates used to create the classifier, there were six different STs (ST11, ST15, ST16, ST258, ST307, and ST340) and seven different KLs (KL102, KL105, KL107, KL151, KL24, KL51, and KL64). Among ST11 isolates, there were 10 KL64 and 8 KL105. All ST15 were KL24 (*n* = 6), ST16 were KL51 (*n* = 18), ST258 were KL107 (*n* = 5), ST307 were KL102 (*n* = 3), ST340 were KL151 (*n* = 4) (Table S1).

**Fig 1:**
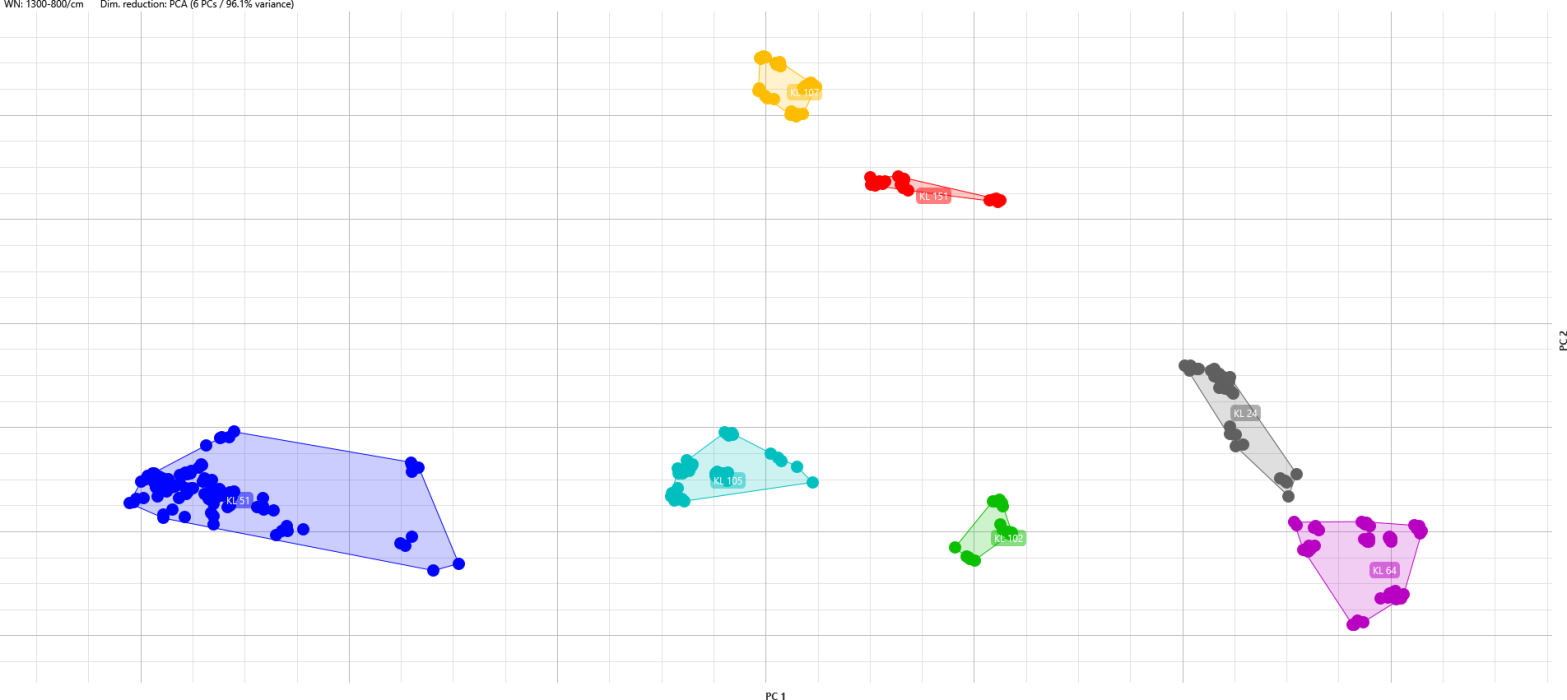
Isolates from the training set grouped by KL. Each color represents one KL type, and each dot represents one spot.

Considering the analysis by KL, all isolates of the training set were grouped according to their KL type (Fig. 1).

**Fig 2:**
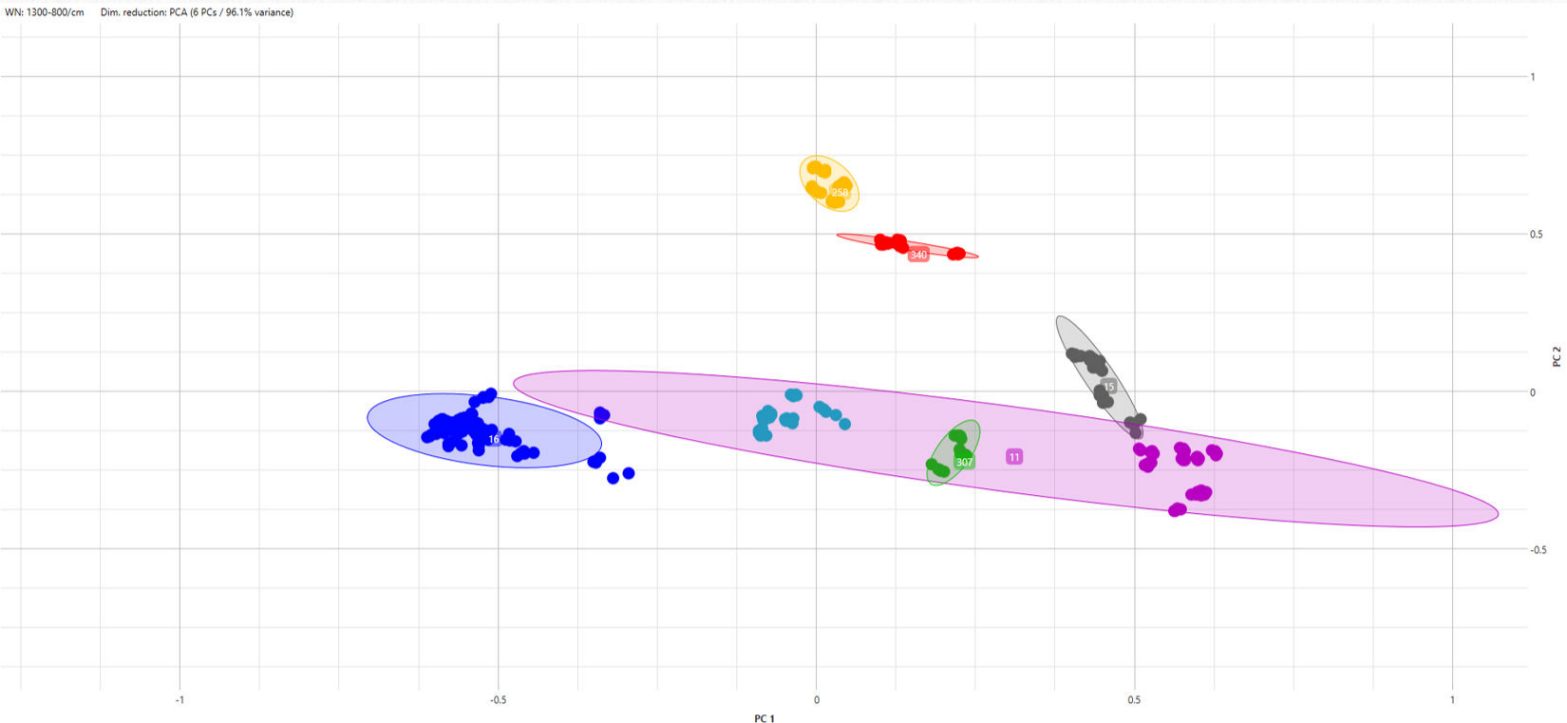
Isolates from the training set grouped by ST. Each dot color represents one KL type, and each dot represents one spot. The ellipses indicate the area of the STs.

When considering the analysis by ST, different KLs were grouped in the same cluster, and ST307 was plotted into the ST11 (Fig. 2).

The remaining isolates (validation set) were classified into a KL using the developed classifier as demonstrated in Table 1.

**TABLE 1:** Isolates from the validation set classified by the KL classifier^*a*^

Isolate	Kl (WGS)	KL (IR Biotyper)	Score classification (IR Biotyper)
GKPN0326	KL 24	KL 24	100%
GKPN0333	KL 105	KL 105	80%
GKPN0308	KL 105	KL 105	100%
GKPN0331	KL 105	KL 105	100%
GKPN0332	KL 64	KL 64	100%
GKPN0273—isolate 1	KL 64	KL 64	100%
GKPN0273—isolate 2	KL 64	KL 64	100%
GKPN0353	KL 64	KL 64	100%
GKPN0375	KL 151	KL 151	**0%**
GKPN0374	KL 151	KL 151	100%
GKPN0358	KL 107	KL 107	100%
GKPN0355	KL 107	KL 107	100%
GKPN0356	KL 107	KL 107	100%
GKPN0322	KL 51	KL 51	100%
GKPN0267	KL 51	KL 51	100%
GKPN0354	**KL 15**	**KL 102**	**0%**
GKPN0328	**KL 46**	**KL 102**	**0%**
GKPN0311	**KL 10**	**KL 102**	**0%**
GKPN0327	**KL 10**	**KL 102**	**0%**

^*a*^ Boldface type indicates disagreement between methods.

**Fig 3:**
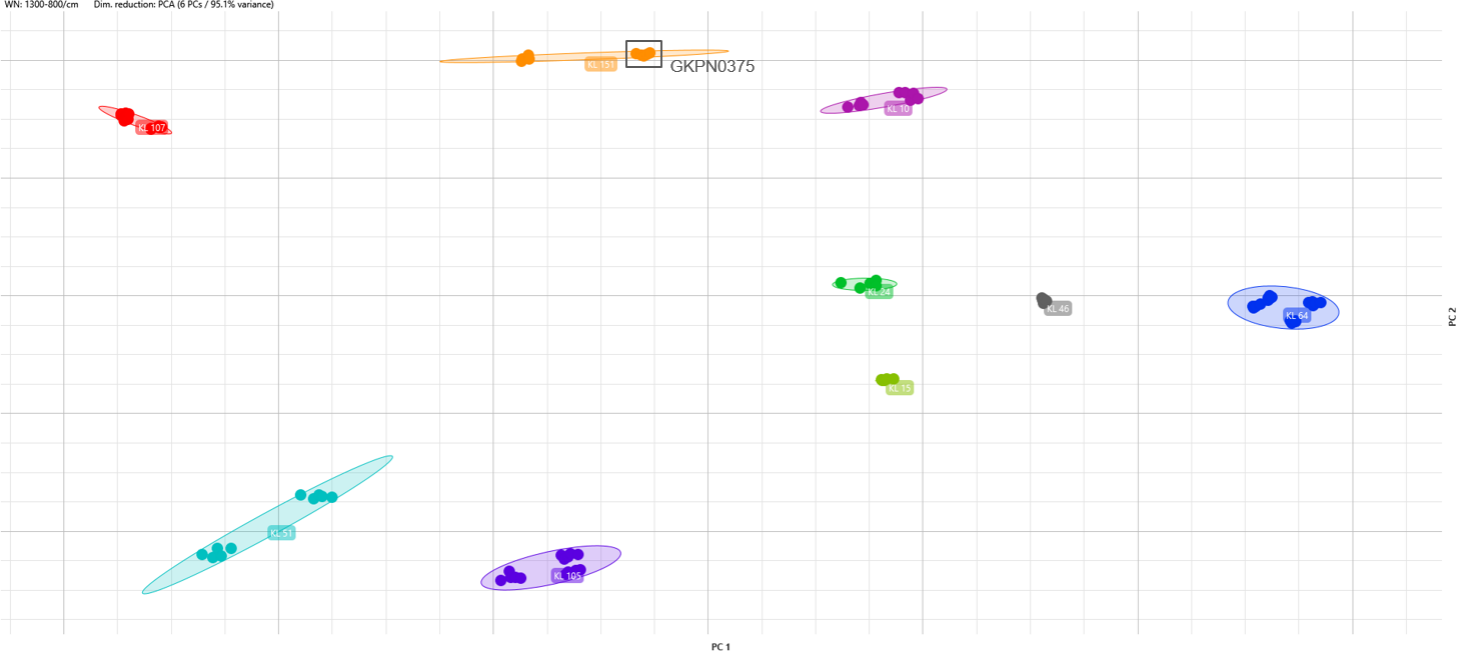
Isolates from the validation set grouped by KL. Each color represents one KL type, and each dot represents one spot. Isolate GKPN0375 (0% score) is marked in the square.

The isolates used to validate the classifier were also plotted into a 2D graphic, which allows to observe the distribution of the isolates according to their KL type as determined by WGS (Fig. 3).

## DISCUSSION

FTIR is a phenotypic method traditionally used in chemistry to determine the molecular composition of a wide variety of structures in a rapid, non-destructive, simple, cheap, and high-throughput manner. FTIR has also evolved for bacterial identification (16). The IR Biotyper equipment (Bruker Daltonik, GmbH), available since 2018, is a system based on the FTIR technology, which allows rapid bacterial molecular typing (8). Until recently, a few studies have evaluated the capability of IR Biotyper to characterize isolates compared to WGS (9, 17–19).

Considering *K. pneumoniae* isolates, recent studies have indicated that the IR Biotyper did not present reliable results to classify isolates in the same cluster of ST. In fact, the results demonstrated that the IR Biotyper grouped in the same cluster isolates of different STs (20) or did not group all the same STs within the same clusters (21). We also found similar results when considering the results of IR Biotyper in comparison to the STs established by WGS.

*K. pneumoniae* is known to have a mucoid appearance in culture media due to well-developed capsules, which are divided into 77 types. Also, capsule typing has been demonstrated to be a good marker for serotyping with good reproducibility and the capability to differentiate clinical isolates (4, 22). Additionally, approximately all capsule serotypes are correlated to the KL (14). We, therefore, attempted to evaluate the capability of IR Biotyper to type *K. pneumoniae* according to the ST as well as KL. As mentioned before, considering only the ST, the IR Biotyper presented a low correlation with WGS, as isolates belonging to the same ST were distributed in different clusters as well as different STs were grouped into the same cluster (Fig. 2). When we use KL to evaluate *K. pneumoniae*, all 73 isolates were grouped according to their KL type (Fig. 1 and 3), indicating that FT-IR correlates well with KL for typing *K. pneumoniae* isolates using the IR Biotyper. However, it is important to notice that this study did not include multiple STs sharing the same KL, which would allow direct comparison of KL clustering across different genetic backgrounds. Therefore, it remains unconfirmed whether FT-IR typing by KL would always distinguish unrelated STs with the same KL, and future studies are needed to address these complementary genomic approaches.

Furthermore, we created a classifier using 54 isolates correctly separated by the KL, in order to classify the remaining isolates. When the classification was performed, 15 isolates were correctly classified according to their KL, whereas only the isolates with KL15, KL10, and KL46 (not included in the classifier training set) were wrongly classified as KL102. Nevertheless, the score of this classification—the confidence value (0–100%) generated by the classifier when assigning a KL type—was 0% (Table 1), which indicates that there is no reliability in this classification, as expected. Interestingly, one isolate (GKPN 0375), which was correctly classified into its KL, showed a 0% score, indicating that the classifier may be improved, possibly considering a different cutoff or different parameters for accepting the results of the classifier. Therefore, more studies are needed before considering the classifier for use in a laboratory routine.

To the best of our knowledge, this is the first study to use the KL to type different strains of *K. pneumoniae* using the IR Biotyper technology. Use of a classifier considering the KLs and STs in the IR Biotyper demonstrated to be a promising tool to type *K. pneumoniae* in a faster and cost-effective way compared to WGS.

## SUPPLEMENTAL MATERIAL

Figure S1 spectrum.01146-25-s0001.docx *Klebsiella pneumoniae* in Mueller-Hinton agar, showing classic and translucent phenotypes.

Table S1 spectrum.01146-25-s0002.xlsx Isolates used for the training set.

Table S2 spectrum.01146-25-s0003.xlsx Isolates used for the validation set.

ASM does not own the copyrights to Supplemental Material that may be linked to, or accessed through, an article. The authors have granted ASM a non-exclusive, world-wide license to publish the Supplemental Material files. Please contact the corresponding author directly for reuse.
