# Erratum for Orlandi Barth et al., “Use of IR Biotyper as a feasible methodology to type *Klebsiella pneumoniae*”

**DOI:** 10.1128/spectrum.04080-25

**Published:** 2026-07-10

**Authors:** Patricia Orlandi Barth, Camila Mörschbächer Wilhelm, Dariane Castro Pereira, Laura Czekster Antochevis, Andreza Francisco Martins, Afonso Luís Barth

## ERRATUM

Volume 13, no. 11, e01146-25, 2025, https://doi.org/10.1128/spectrum.01146-25. In Table S2, the correct ST of the isolate GKPN0308 is “ST11,” not “ST258”. The correct Table S2 is provided in this erratum.

## SUPPLEMENTAL MATERIAL

TABLE S2 spectrum.04080-25-s0001.xlsx Isolates used for the validation set.

ASM does not own the copyrights to Supplemental Material that may be linked to, or accessed through, an article. The authors have granted ASM a non-exclusive, world-wide license to publish the Supplemental Material files. Please contact the corresponding author directly for reuse.

