# Supplementary material for: Use of IR Biotyper as a feasible methodology to type Klebsiella pneumoniae
Source: Microbiol Spectr. 2025 Oct 6;13(11):e01146-25. doi: 10.1128/spectrum.01146-25 (PMC12584611; doi:10.1128/spectrum.01146-25)
Supplement: Figure S1 — Klebsiella pneumoniae in Mueller-Hinton agar, showing classic and translucent phenotypes. [file spectrum.01146-25-s0001.docx]

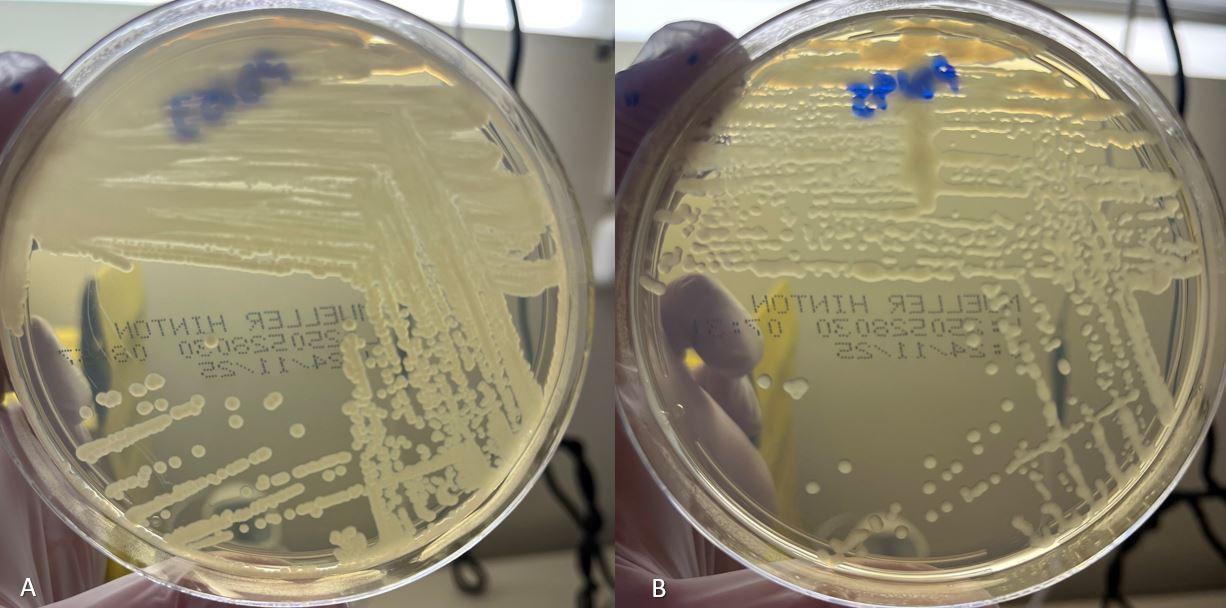


**Sup. Fig.1:** A = *Klebsiella pneumoniae* in Agar Müeller Hinton, with classic phenotype (not translucent), used in IR Biotyper. B = *Klebsiella pneumoniae* in Agar Müeller Hinton, with translucent phenotype, which were excluded for IR Biotyper analysis.
